# Force decay evaluation of latex and non-latex orthodontic intraoral elastics: *in vivo* study

**DOI:** 10.1590/2177-6709.23.6.042-047.oar

**Published:** 2018

**Authors:** Daniela Ferreira de Carvalho Notaroberto, Mariana Martins e Martins, Maria Teresa de Andrade Goldner, Alvaro de Moraes Mendes, Cátia Cardoso Abdo Quintão

**Affiliations:** 1 Universidade do Estado do Rio de Janeiro, Programa de Pós-graduação em Odontologia, Departamento de Odontologia Preventiva e Comunitária (Rio de Janeiro/RJ, Brazil).; 2 Universidade Federal Fluminense, Faculdade de Odontologia, Disciplina de Ortodontia (Niterói/RJ, Brazil).; 3 Universidade do Estado do Rio de Janeiro, Departamento de Odontologia Preventiva e Comunitária, Disciplina de Ortodontia (Rio de Janeiro/RJ, Brazil).

**Keywords:** Elastomers, Tensile strength, Latex, Silicone elastomers.

## Abstract

**Objective:**

This clinical study was conducted in order to evaluate force decay over time of latex and non-latex orthodontic intraoral elastics.

**Methods:**

Patients (n = 15) were evaluated using latex and non-latex elastics in the periods of : 0, 1, 3, 12 and 24 hours. The rubber bands were transferred to the testing machine (EMIC DL-500 MF), and force values were recorded after stretching the elastic to a length of 25mm. Paired *t* test was applied and analysis of variance (ANOVA) was used to evaluate the variation of force generated. LSD (Fisher’s least significant difference) *post-hoc* test was thus employed.

**Results:**

As regards the initial forces (zero time), the values of force for non-latex elastic were slightly higher than for the latex elastic. In the subsequent times, the forces generated by the latex elastic showed higher values. Regarding the material degradation, at the end of 24 hours the highest percentage was observed for non-latex elastic.

**Conclusions:**

The latex elastics had a more stable behavior during the studied period, compared with non-latex.

## RESUMO

**Objetivo:**

o objetivo deste estudo clínico foi avaliar e comparar o comportamento dos elásticos de látex e não látex quanto à perda de força ao longo do tempo.

**Método:**

os pacientes (n = 15) foram avaliados usando ambos os tipos de material (látex e não látex) em cada tempo: 0, 1, 3, 12 e 24 horas. Os elásticos foram transferidos para a máquina de ensaios mecânicos (EMIC DL-500 MF) e os valores de força foram registrados após a distensão dos elásticos a uma distância de 25 mm. Foi aplicado o teste *t* pareado, e a análise de variância (ANOVA) foi realizada para verificar a variação das forças geradas em todos os tempos estudados. Em seguida, o teste *post-hoc* LSD (Fisher’s *least significant difference*) foi aplicado.

**Resultados:**

quanto às forças iniciais (tempo zero), os valores de força dos elásticos não látex foram ligeiramente maiores do que dos elásticos de látex. Nos tempos subsequentes, as forças geradas pelos elásticos de látex apresentaram valores superiores às geradas pelos elásticos não látex. Em relação à degradação do material, ao fim do período de 24 horas, o maior percentual foi observado pelos elásticos não látex.

**Conclusões:**

os elásticos de látex apresentaram comportamento mais estável durante o período de estudo, em comparação aos não látex.

## INTRODUCTION

Orthodontic elastics are still valuable devices, widely used in clinical practice, because they present many varieties of application regarding the direction of force applied to the teeth to be moved, thus helping in the correction of several malocclusions.^1^

Initially, these elastics were composed of natural rubber (latex), a raw material discovered and used for centuries by the ancient Inca and Mayan civilizations.^2^ They are still widely used today,^3^ ^,^ ^4^ mainly because of the high flexibility and low cost.^5^ However, by the 1980s, allergic reactions to latex became more prevalent and better recognized.^6^ ^,^ ^7^ With the aim of maintaining the mechanical properties of the elastics, without causing allergy in patients with hypersensitivity to latex, orthodontic rubber elastics based on synthetic rubber (non-latex) have been used more frequently.^8^ ^-^ ^10^ So, it is imperative to evaluate and compare the mechanical properties of these two different materials.

Some laboratory studies were performed to analyze the behavior of non-latex elastics compared to latex elastics.^4^ ^,^ ^6^ ^,^ ^9^ ^,^ ^11^ ^-^ ^13^ Most of these studies showed a marked reduction in the strength levels of these elastics within the first 24 hours, showing the non-latex elastics limitation in maintaining a constant force for an extended period.^4,6,11,12^ Manufacturers have added chemical substances to retard these effects and extend the lifetime of these elastomers.^2^

However, in the oral cavity, the characteristics of elastics materials are affected by physical, chemical and biological factors, some of them related to functional activities, salivary changes and nutrition habits.^3^ ^,^ ^14^ Non-latex elastics also must be tested in the oral environment and, at our knowledge, just one clinical study^15^ was reported in the literature. The related article did not evaluate the first hours of use, which are described as being critical in relation to the greatest force loss of the intermaxillary elastics. Other few clinical studies evaluating intraoral elastics were performed evaluating only latex elastics.^3^ ^,^ ^16^

Thus, the purpose of the present study was to evaluate *in vivo* the force degradation of latex and non-latex elastics exchanged at different times, over a period of 24 hours. 

## MATERIAL AND METHODS

A prospective controlled clinical trial with split-mouth design was conducted to evaluate the behavior of latex and non-latex elastics over 24 hours.

This study was approved by the Research Ethics Committee of Hospital Universitário Pedro Ernesto/UERJ (Ethics Committee document #285.772). All participants received prior information about the research and signed an informed consent form.

Intraoral latex (*n*= 75) and non-latex elastics (*n*= 75) (American Orthodontics, Sheboygan, USA), at a 3/16-inch size were tested. They were within the expiration dates and stored in sealed plastic packages in a cool and dark environment. 

Using a specific formula for split-mouth or crossover studies,^17^ sample size calculation was performed based in a pilot study (*n*= 5), in which the values in gram-force (gf) generated by the elastics of the five patients were used. The sample size of the present study was then determined to be 13 patients, with 80% of test power, 5% of alpha level, 24.75 of standard deviation of difference, and 20 of average difference; however, to avoid missing data, 15 patients were selected for the study. 

Systematic convenience sampling was used, in which participants were selected in a post-graduate orthodontic program of a public university, following dental appointment schedules between February 2016 and August 2016.

Patients (*n*= 15) with mean age of 20.16 years, who were undergoing orthodontic treatment were selected. As inclusion criteria they should be in final phase of the treatment, using rectangular or round arches of 0.020-inch of diameter, with no extractions and with a prescription for using Class II or Class III intermaxillary elastics, on both side of the mouth.

**Figure 1:**
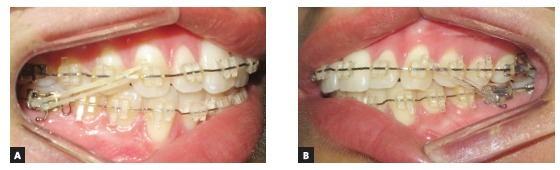
A) Latex elastic on the right side; B) non-latex elastic on the left side.

The side selection for the use of each elastic material (latex or non-latex) was randomized and sequential, using sealed brown envelopes, so that the patient #1 would use latex on the right side and non-latex on the left side (Fig 1), patient #2 would use non-latex on the right side and latex on the left side, and so on. 

The elastics were attached to canine and first molars hooks (Fig 1). The mean value of the distance between the hooks for the placement of the elastic was 25 mm. The patients were instructed to use an intermaxillary elastic for 1, 3, 12 and 24 hours. They could only remove the elastic to eat or brush the teeth, replacing the same elastic then. 

By the time of elastic removal, the patient was referred to the clinic next to the laboratory, allowing the elastics to be removed from the patient mouth and immediately adapted to the mechanical testing machine (EMIC DL-500 MF), for force measurement. Each elastic was carefully transferred with a pair of tweezers by the same operator from the patient’s mouths to the test machine, and was then discarded after measurement.

The cross-head speed of the testing machine was 30 mm/min, as recommended by Fernandes et al^18^ and Lopez et al,^12^ and the calibrated load cell capacity was 2.0 Kgf. Extension force magnitudes of the elastics were immediately recorded after they were removed from the patient’s mouth and stretched at a distance of 25 mm. All procedures were performed by the same operator.

Descriptive statistics were used as mean, median, standard deviation, maximum and minimum, relative to the elastic force values measured in grams/force and organized for the amounts of liberated force observed at different time intervals.

The collected data were analyzed by paired *t* test, in order to compare the different types of elastic at each time; and by analysis of variance (ANOVA), to evaluate the variation of the forces generated at all selected times. A *post-hoc* test (Fisher’s least significant difference, LSD) was applied to identify which pairs of the force remained significantly different during the study (SPSS software version 20.0; IBM, Armonk, NY). A *p* value less than 0.05 was considered statistically significant.

## RESULTS

Although in baseline (control group) non-latex elastics have generated higher values than latex elastics when stretched to 25mm, in all the other periods the latex strength force values were superior to non-latex elastics. Paired *t* test showed significant difference between latex and non-latex elastics in almost all observed times, except in baseline (control group) (Table 1). 

**Table 1:** Mean and standard deviation of the forces (gf) generated by intermaxillary orthodontic latex and non-latex elastics, according to time of experiment.

	Time				
Type of elastic	0h	1h	3h	12h	24h
Latex	224.49 ± 11.09^a^	191.70 ± 11.92^b^	186.18 ± 10.25^bc^	179.13 ± 10.41^c^	179.75 ± 16.45^c^
Non-latex	228.03 ± 13.33^a^	165.72 ± 10.19^b^	162.43 ± 13.68^b^	146.43 ± 13.27^c^	138.56 ± 14.14^d^
Paired t test	p = 0.470	p < 0.001	p < 0.001	p < 0.001	p < 0.001

Values with different superscript letters (a, b, c, d) indicate significant differences, over time (LSD post-hoc test).

Analysis of variance for paired data (ANOVA) detected significant differences when comparing the strength force values of latex and non-latex elastics between all times studied (*p*< 0.001). Then, LSD *post-hoc* test was performed and statistical differences were found (Table 1). 

**Figure 2:**
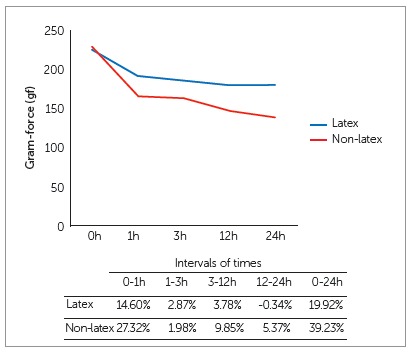
Latex and non-latex elastics behavior in the 24-hour period.

Force degradation percentages for latex and non-latex elastics, between all the times are shown in Figure 2. The highest percentage difference generated of force decay occurred between baseline and 1 hour (14.60% for latex elastics and 27.32% for non-latex elastics). Over the next intervals (1-3 hours; 3-12 hours and 12-24 hours), the percentage difference generated of force decay occurred more subtly. After 24 hours of the study, the biggest difference between the degradation percentage of the force was observed for non-latex elastics (39.23%) compared to latex elastics (19.92%).

All the participants had an excellent cooperation with the use of elastic, but of the 15 evaluated patients, 7 needed to repeat the use of the elastics during the 24-hour period, due to the rupture of the non-latex elastics. 

## DISCUSSION

The literature has provided several studies evaluating the force released by the intermaxillary elastics conducted in laboratorial environment.^5^ ^,^ ^6^ ^,^ ^8^ ^-^ ^13^ ^,^ ^18^ ^-^ ^21^ Some have evaluated the differences between the forces released by latex and non-latex elastics.^4^ ^,^ ^6^ ^,^ ^9^ ^,^ ^11^ ^-^ ^13^ However, it is known that the oral medium is much more complex, with a great variety of interacting factors such as salivary pH, diet, oral hygiene conditions and oral habits.^3^ ^,^ ^14^ *In situ* study, as conducted in this research, is the more precise method to test materials that will be held in the oral environment. A split-mouth study model was adopted, reducing variability and allowing a smaller sample.^17^

The elastic force was measured at 0 (baseline), 1, 3, 12 and 24 hours, considering the fact that laboratory studies indicate the greatest force drop occurring in the first hours.^5^ ^,^ ^18^ ^,^ ^19^ ^,^ ^22^ ^,^ ^23^ The only clinical study found in our literature review that analyzed the differences between the forces released by the latex and non-latex elastics was conducted by Pithon et al^15^ and evaluated only 0, 12 and 24 hours. The total time of 24 hours was chosen because this is the period in which routinely it is asked the patient to replace the elastics by new ones.

The results showed that both elastics (latex and non-latex) have progressive force reductions over time (24 hours period).

The biggest drop of the force unleashed by the latex elastics occurred in the first hour, with significant difference. On subsequent times, the decrease in strength was softer, without statistical significance (Table 1). These results are in agreement with the findings of laboratory studies affirming that the greatest fall in the values of the forces generated by the latex intermaxillary elastics occur in the first hours after their distension and as time progressed, the degradation became slower.^5^ ^,^ ^18^ ^,^ ^19^ ^,^ ^24^ The few clinical studies conducted also reported this behavior. Wang et al^3^ and Qodcieh et al^16^ found that the large force loss occurred within the first hour. 

The non-latex elastics also demonstrated a significant large decrease in the amount of force generated between 0 and 1 hour, but continued to show significant loss of force within 3 to 12 hours and within 12 to 24 hours (Table 1). Similarly, Kersey et al,^4^ in a study involving the non-latex intermaxillary elastics of 1/4-in diameter, noticed a decrease in the values of forces generated between 20% and 30% in the first hour, and 40% to 60% after 24 hours. However, higher percentage values than those obtained in this study were reported by Araujo and Ursi,^23^ who observed a reduction in the amount of force generated by the non-latex elastics from 20.31% to 38.47% in the first hour, and from 47.7% to 75.95% on 28 days of stretching. The only clinical study with non-latex elastics did not evaluate the first hours, but showed a progressive and significant reduction of the force generated by these elastics from 0 to 12 hours and also from 12 to 24 hours.^15^

When the forces generated by the intermaxillary elastics of the two types (latex and non-latex) were compared, significant differences were found in all the times studied, except for the baseline (Table 1). These data are in agreement with the study by Pithon et al,^15^ who found that latex intermaxillary elastics with 1/8-in diameter lose less force over time compared to non-latex elastics. However, in the study of Pithon et al,^15^ latex and non-latex elastics 1/4-in and 5/16-in in diameter demonstrated no significant differences after 24 hours. 

The most significant decrease in force values ​​occurred in the first hour, for both latex and non-latex elastics, with the difference percentage higher for non-latex elastic, of 27.32%, compared to the difference for latex, 14.60%. After 24 hours, the percentage difference for non-latex elastics was 39.23% and for latex was 19.92% (Fig 2). The laboratory studies^9^ ^,^ ^12^ found similar results, detecting greater loss of strength for the non-latex elastics, when compared to the latex ones. Kersey et al^4^, when comparing latex and non-latex elastics from a single manufacturer (American Orthodontics, the same manufacturer used in this study), found that latex elastics maintain higher strength levels over 24 hours, retaining 83% of initial strength, compared to 69% retained by non-latex elastics. The clinical study showed the same results, a greater loss of the initial force in 24 hours for the non-latex elastics.^15^

Of the 15 patients evaluated, 7 needed to repeat the use of the elastics during the 24-hour period, due to the rupture of the non-latex elastics. This limitation of non-latex elastics was also observed in the studies of Russell et al^6^ and Hwang and Cha.^13^ No fracture was observed in latex elastic throughout the clinical study.

These findings are important because non-latex elastics are an alternative for patients with latex sensitivity. It is necessary to understand the clinical behavior of these elastics in order to establish the best way to use them. As the clinical behavior was different at all times tested in the oral cavity (1, 3, 12, 24 hours) and having these non-latex elastics released smaller forces and losing greater amount of force over time, it is suggested that the non-latex elastics must be changed more frequently in order to obtain a better action during their use in orthodontic treatment. 

It is important to emphasize that this study evaluated the difference in composition between elastics. Thus, only one trademark and one size were evaluated, for a better interpretation of the results. Other brands and diameters may perform differently and must be tested. 

## CONCLUSIONS

Latex elastics showed a more stable behavior within 24 hours, when compared to non-latex elastics.

During the oral experimental time (3, 12 and 24 hours), the latex elastics had higher force released values, when compared to non-latex elastics.

## References

[1] Singh VP, Pokhrael PR, Pariekh K, Roy DK, Singla A, Biswas KP (2012). Elastics in orthodontics a review. Health Renaissance.

[2] Baty DL, Storie DJ, Von Fraunhofer JA (1994). Synthetic elastomeric chains: a literature review. Am J Orthod Dentofacial Orthop.

[3] Wang T, Zhou G, Tan X, Dong Y (2007). Evaluation of force degradation characteristics of orthodontic latex elastics in vitro and in vivo. Angle Orthod.

[4] Kersey ML, Glover KE, Heo G, Major PW (2003). A comparison of dynamic and static testing of latex and nonlatex orthodontic elastics. Angle Orthod.

[5] Kanchana P, Godfrey K (2000). Calibration of force extension and force degradation characteristics of orthodontic latex elastics. Am J Orthod Dentofacial Orthop.

[6] Russel KA, Milne AD, Khanna RA, Lee JM (2001). In vitro assessment of the mechanical properties of latex and non-latex orthodontic elastics. Am J Orthod Dentofacial Orthop.

[7] Hain MA, Longman LP, Field EA, Harrison JE (2007). Natural rubber latex allergy implications for the orthodontist. J Orthod.

[8] Hanson M, Lobner D (2004). In vitro neuronal cytotoxity of latex and nonlatex orthodontic elastics. Am J Orthod Dentofacial Orthop.

[9] Aljhani AS, Aldrees AM (2010). The effect of static and dynamics testing on orthodontic latex and non-latex elastics. Orthod Waves.

[10] Alavi S, Tabatabaie AR, Hajizadeh F, Ardekani AH (2014). An In-vitro comparison of force loss of orthodontic non-latex elastics. J Dent (Tehran).

[11] Kamisetty SK, Nimagadda C, Begam MP, Nalamotu R, Srivastav T, Shwetha GS (2014). Elasticity in Elastics-An in-vitro study. J Int Oral Health.

[12] López N, Vicente A, Bravo LA, Calvo JL, Canteras M (2012). In vitro study of force decay of latex and non-latex orthodontic elastics. Eur J Orthod.

[13] Hwang CJ, Cha JY (2003). Mechanical and biological comparison of latex and silicone rubber bands. Am J Orthod Dentofacial Orthop.

[14] De Genova DC, McInnes-Ledoux P, Weinberg R, Shaye R (1985). Force degradation of orthodontic elastomeric chains-a product comparison study. Am J Orthod.

[15] Pithon MM, Mendes JL, Silva CA, Santos RL, Coqueiro RD (2016). Force decay of latex and non-látex intermaxillary elastics a clinical study. Eur J Orthod.

[16] Qodcieh SMA, Al-Khateeb SN, Jaradat ZW, Abu Alhaija ESJ (2017). Force degradation of orthodontic latex elastics An in-vivo study. Am J Orthod Dentofacial Orthop.

[17] Pandis N (2012). Sample calculation for split-mouth designs. Am J Orthod Dentofacial Orthop.

[18] Fernandes DJ, Fernandes GMA, Artese F, Elias CN, Mendes AM (2011). Force extension relaxation of medium force orthodontic latex elastics. Angle Orthod.

[19] Bishara SE, Andreasen GF (1970). A comparison of time related forces between plastics alastiks and latex elastics. Angle Orthod.

[20] Sauget PS, Stewart KT, Katona TR (2011). The effect of pH levels on non-latex latex interarch elastics. Angle Orthod.

[21] Santos RL, Pithon MM, Romanos MTV (2012). The influence of pH levels on mechanical and biological properties of nonlatex and latex elastics. Angle Orthod.

[22] Oesterle LJ, Owens JM, Newman SM, Shellhart WC (2012). Perceived vs measured forces of interarch elastics. Am J Orthod Dentofacial Orthop.

[23] Araújo FBC, Ursi WJS (2006). Study of the degradation of the force generated by synthetic orthodontic elastics. Rev Dental Press Ortod Ortop Facial.

[24] Moris A, Sato K, Facholli AFL, Nascimento JE, Sato FRL (2009). In vitro study of the strength degradation of latex orthodontic elastics under dynamic conditions. Rev Dental Press Ortod Ortop Facial.

